# 仪器分析自主实验设计：在线固相萃取-高效液相色谱-柱后衍生测定蔬菜中痕量农药残留

**DOI:** 10.3724/SP.J.1123.2025.10020

**Published:** 2026-09-08

**Authors:** Yan LI, Yuntao LIU, Xien HUANG, Ziyi ZHOU, Ziqiu WANG, Jing WANG, Fei DING, Jing NAN, Anna TANG

**Affiliations:** 南开大学化学学院，化学国家级实验教学示范中心，天津 300071; College of Chemistry，Nankai University，National Demonstration Center for Experimental Chemistry Education，Tianjin 300071，China

**Keywords:** 自主设计实验, 在线固相萃取, 高效液相色谱, 柱后衍生, 农残分析, self-designed experiment, online solid-phase extraction （online SPE）, high performance liquid chromatography （HPLC）, post-column derivatization, pesticide residue analysis

## Abstract

为顺应国家现代教育理念及创新型人才培养的要求，仪器分析实验课程在常规实验的基础上增设了学生自主设计实验环节。这一改革旨在强化学生“学以致用”能力的培养，使学生在掌握仪器操作技能的同时，能预判和解决实验过程中所遇到的实际问题。在教师的指导下，学生自主搭建了在线固相萃取-液相色谱分离-柱后衍生荧光检测装置，建立了蔬菜样品中痕量氨基甲酸酯类农药的检测方法，并通过实践攻克了提升方法灵敏度与精密度、消除基质干扰、实现快速分析等关键技术难题。该自主设计实验从学生的学习特点和兴趣出发，有效激发了学生的学习主动性和创新潜能，显著提升了教学效果与学习效率。

## Abstract (en)

In line with modern educational concepts and the demand for innovative talents， a course focused on self-designed experiments has been incorporated into the traditional instrumental analysis curriculum. This initiative aims to enhance students’ ability to apply knowledge effectively. While mastering instrument operation， students are encouraged to anticipate potential challenges during experiments. Under instructor guidance， students constructed an online system for solid-phase extraction coupled with liquid chromatography and post-column derivatization with fluorescence detection. They addressed real analytical needs such as improving sensitivity and precision， minimizing interference， and conducting rapid analyses of trace carbamate pesticides in vegetable samples. This self-designed liquid chromatography experiment course， tailored to students’ learning characteristics and interests， successfully engages their initiative and fosters independent innovation capabilities. As a result， it also improves teaching effectiveness and enhances learning efficiency.

南开大学化学学院仪器分析实验课程面向多学院中、高年级本科生开设，涵盖了光谱、波谱、色谱等十余种分析测试仪器，旨在帮助学生掌握常用的分析手段，为科学研究奠定基础。传统的仪器分析实验课程存在内容割裂、操作模式单一等问题，限制了学生系统训练与创新能力的培养。为此，近年来各高校陆续推进仪器分析实验课程改革来完善课程建设^［1-4］^。南开大学化学实验教学示范中心也在原有课程体系中引入了“仪器分析实验课程自主设计实验”环节，该环节以社会关注的实际问题为导向，引导学生开展从选题设计到实验实施的完整科研训练^［5］^。本文以果蔬中氨基甲酸酯类农药残留检测为例，体现该教学模式下学生综合运用多种分析方法解决实际问题的过程，为课程改革提供了可借鉴的实施案例。

农药残留是食品安全领域的社会热点问题。氨基甲酸酯类农药自20世纪被开发以来，曾广泛应用于果蔬病虫害防治^［6］^，通过抑制昆虫乙酰胆碱酶和羧酸酯酶的活性，达到杀虫的效果^［7］^。然而其对人类神经系统也具有潜在毒性，长期摄入会引发慢性健康风险；此外，农药残留对生态环境的污染也呈现系统性特征^［8］^，严重威胁区域的生态安全，因此我国对蔬菜和水果中氨基甲酸酯类农药残留设有严格的限量标准（部分含量≤0.02 mg/kg）^［9］^。

目前检测氨基甲酸酯类农药残留的方法，主要有液相色谱法和液相色谱-质谱法^［10］^。高效液相色谱法属于本科实验中的教学内容。氨基甲酸酯类农药，具有较强的疏水性。实验中选用具有疏水性的十八烷基硅烷键合硅胶（ODS）作为反相色谱分离固定相，甲醇和水组成流动相，通过梯度洗脱程序进行分离检测。相较传统教学的等度分离方法，学生通过对梯度分离方法的优化，加深了对反相色谱理论知识的理解。

针对植源性样品基质复杂、农残含量低的问题，提高测定方法的灵敏度、减少杂质干扰成为设计实验方案的重要考虑因素。SPE作为一种高效的富集手段，广泛应用于农残检测。近些年，已有多种吸附材料用于痕量农药组分的富集^［11-15］^。多孔聚（苯乙烯-二乙烯基苯）（PS-DVB）微球，具有比表面积大、传质能力强、机械强度高、稳定性好以及成本低等优点。实验中选用PS-DVB作为SPE材料。PS-DVB除了疏水表面与被分析物之间的疏水作用外，其苯环上的*π*电子能够与被分析物的芳环和双键上的*π*电子相互作用，因此SPE材料对这3种代表性的氨基甲酸酯类化合物具有较强的吸附能力，而对非芳香性干扰杂质的保留弱，从而实现了被分析物和干扰杂质的选择性富集、分离。

氨基甲酸酯类农药本身无荧光。本实验采用柱后衍生荧光法，使氨基甲酸酯水解生成的一级胺与邻苯二甲醛（OPA）和巯基乙醇反应，生成具有刚性平面结构的强荧光异吲哚衍生物，使其可以在330 nm的激发波长（*λ*_ex_）和465 nm的发射波长（*λ*_em_）下被灵敏检测。通过该实验加深了学生对荧光分析法理论知识的理解，同时培养学生灵活运用理论知识的能力。

综上，我们建立了在线固相萃取-高效液相色谱-柱后衍生荧光检测（online SPE-HPLC-PCD-FLD）的方案。整个操作和分析流程完全在线化，通过连接富集、洗脱、分离、水解、衍生等各种不同的模块，显著缩短分析时间，提高自动化程度减少人为操作因素带来的误差，特别适用于样品基质复杂的植源性样品中痕量氨基甲酸酯农药残留的检测。

## 1 实验部分

### 1.1 仪器、试剂与材料

Agilent 1260 Infinity Ⅱ高效液相色谱系统，配备4台四元输液泵、手动进样器、柱温箱、荧光检测器（Agilent，美国）；KQ-100超声波清洗器（昆山超声仪器有限公司）； PHSJ-3F实验室pH计（上海仪电科学仪器股份有限公司）；BP2215分析天平（Sartorius，德国）；Milli-Q超纯水系统（Millipore，美国）；加热控制器（实验室自制）。

氢氧化钠、四硼酸钠、氯化钠、浓盐酸均为分析纯，购于天津光复精细化工研究所；OPA、巯基乙醇试剂购于上海阿拉丁生化科技股份有限公司；乙腈、甲醇均为色谱纯，购于天津市康科德科技有限公司；农药标准品购于北京伊诺凯科技有限公司。实验用水为超纯水（电阻率为18.2 MΩ·cm）。蔬菜样品购自当地市场。

OPA衍生试剂：准确称取0.05 g OPA，加入10 mL甲醇，溶解后再加入0.5 g巯基乙醇溶解，用4 g/L 四硼酸钠溶液定容至500 mL，混匀，避光条件下于4 ℃保存。

准确称取10 mg各农药标准品，用甲醇溶解并分别定容至10 mL，配制成1 mg/mL的农药标准溶液。

### 1.2 样品前处理

准确称取10 g试样，加入20 mL乙腈，匀浆提取2 min，提取液过滤至装有3 g NaCl的比色管中，剧烈振荡1 min，在室温下静置30 min。取上清液5 mL，用氮气吹至近干，加入1 mL甲醇溶解残余物，加入水定容至10 mL。

### 1.3 在线SPE-HPLC-PCD-FLD条件

在线SPE-HPLC-PCD-FLD系统如图1所示，采用PS-DVB柱（50 mm×4.6 mm，10 μm）作为在线SPE柱，样品经泵1输送至六通阀中，流经SPE柱富集农残组分。采用ZORBAX SB-C18柱（250 mm×4.6 mm，5 μm）作为分离柱，流动相经泵2输送至六通阀中，平衡色谱分离柱。当样品完成富集过程后，清洗液经泵1清洗预柱。完成清洗后，切换六通阀，流动相经泵2洗脱预柱上富集的农残组分，并在色谱柱中分离。水解液经泵3输送至管路，氨基甲酸酯类农药在100 ℃的条件下充分水解。流出的产物在室温下与泵4输送至管路中的衍生液反应，衍生物由FLD进行荧光信号的检测与分析。

**图1:**
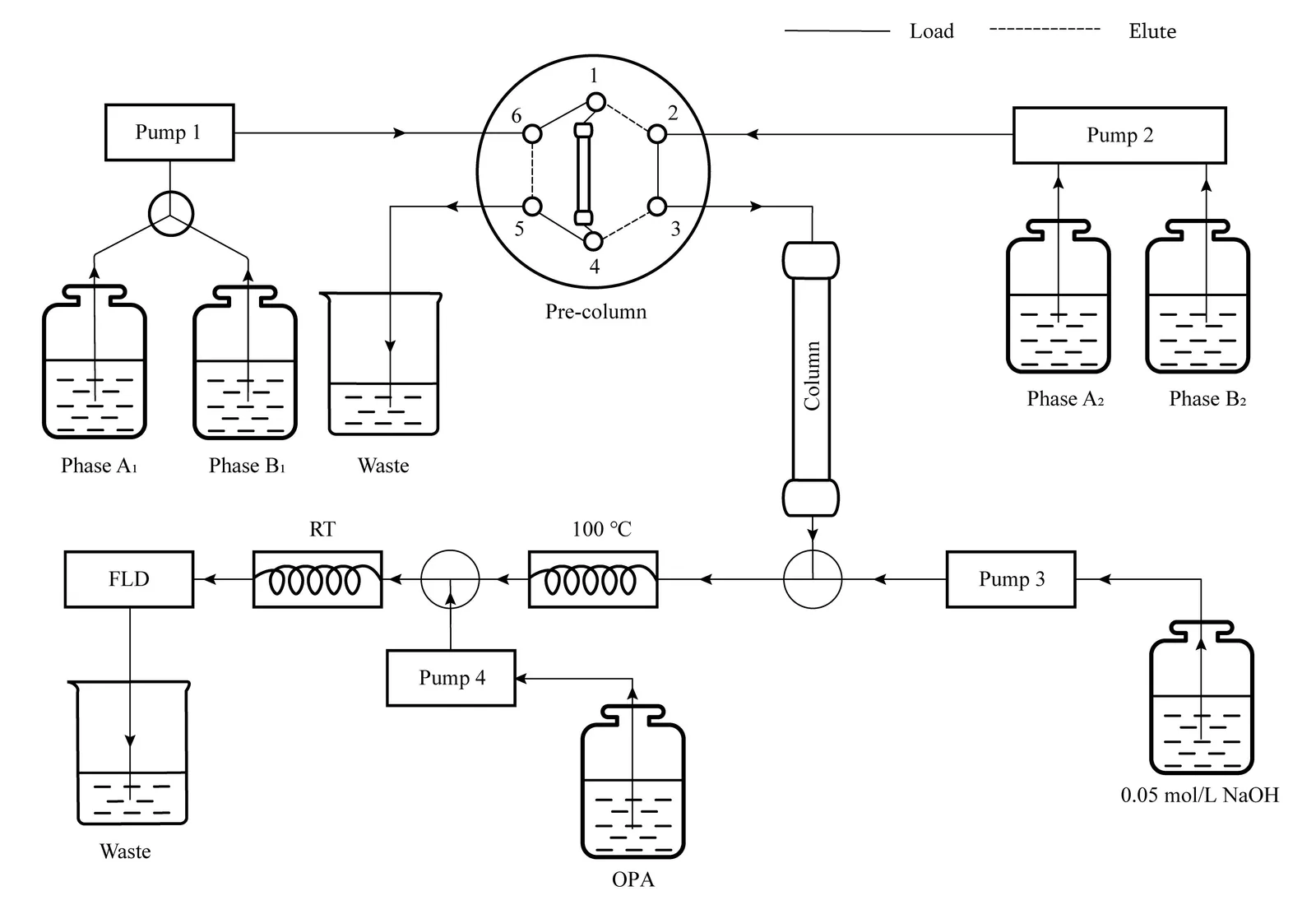
在线SPE-HPLC-PCD-FLD系统

泵1流动相A_1_：样品溶液，流动相B_1_：10%甲醇水溶液，流速0.3 mL/min；泵2流动相A_2_：60%甲醇水溶液，流动相B_2_：甲醇，柱温30 ℃，流速0.8 mL/min；泵3水解液为0.05 mol/L NaOH溶液，水解温度100 ℃，流速0.4 mL/min；泵4衍生液为OPA衍生试剂，衍生温度为室温，流速0.4 mL/min。荧光检测*λ*_ex_=330 nm，*λ*_em_=465 nm。具体流程如表1所示。

**表1:** SPE-HPLC-PCD-FLD程序及阀切换时间表

Stage	Sample time/min	Pump 1		Elution time/min	Pump 2		Valve state
		*φ*（A_1_）/%	*φ*（B_1_）/%		*φ*（A_2_）/%	*φ*（B_2_）/%	
Balance	0	0	100	-	100	0	load
	3.0	0	100	-	100	0	load
Sample loading	3.1	100	0	-	100	0	load
	13.0	100	0	-	100	0	load
Washing	13.1	0	100	-	100	0	load
	14.0	0	100	-	100	0	load
Elution & detection	-	0	0	0	100	0	inject
	-	0	0	8.0	100	0	inject
	-	0	0	12.0	25	75	inject
	-	0	0	24.0	25	75	inject
	-	0	0	25.0	100	0	inject

## 2 结果与讨论

### 2.1 衍生原理及过程

氨基甲酸酯类农药的甲氨基团可以在碱液的作用下生成甲胺，进而与OPA衍生试剂反应生成具有刚性平面结构的强荧光物质，在激发波长330 nm、发射波长465 nm条件下可以检测其荧光信号。该方法也是检测氨基甲酸酯类农药最有效且灵敏的方法之一，原理如图2所示。

**图2:**
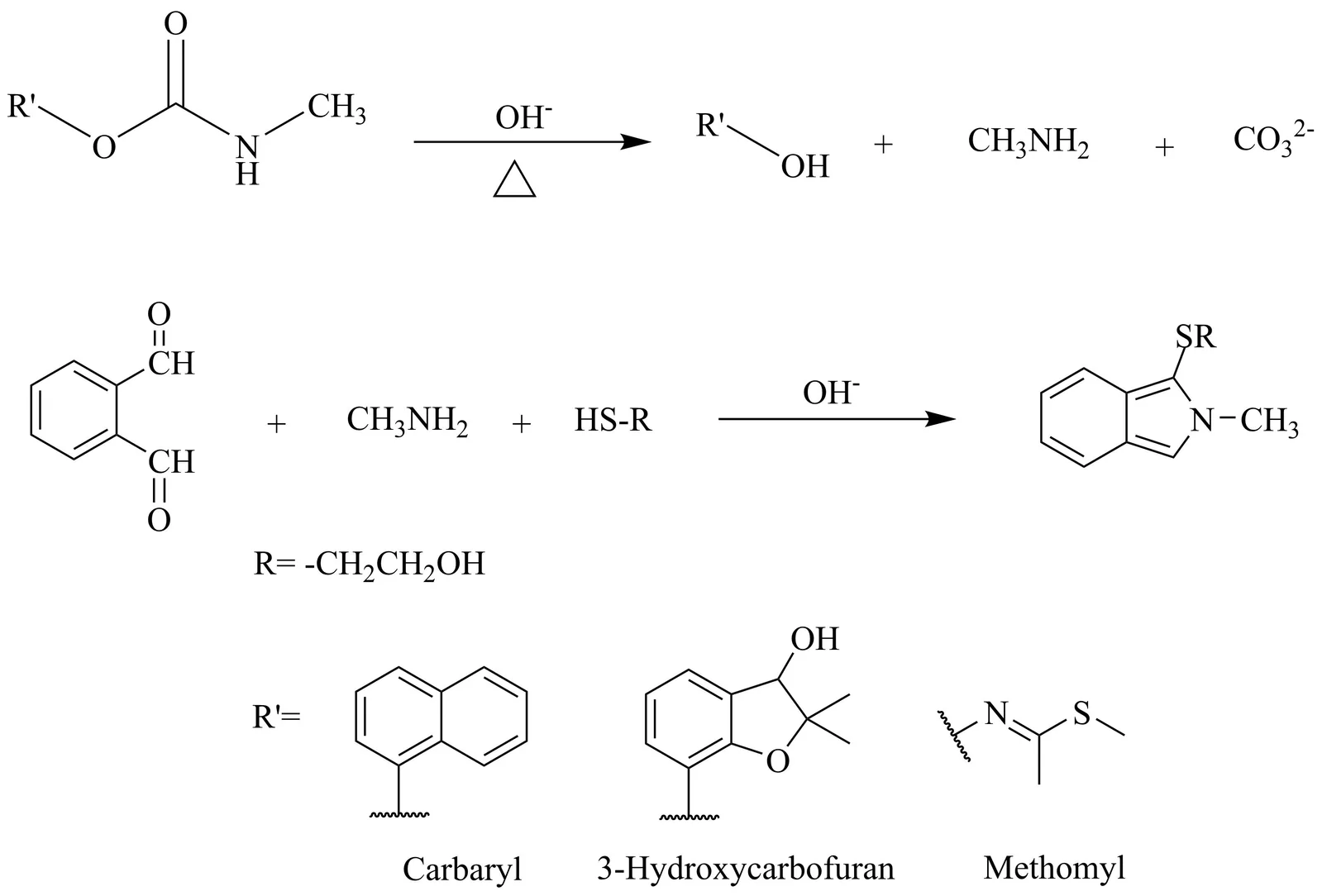
衍生原理

### 2.2 吸附条件优化

分别用盐酸和氢氧化钠将样品溶液pH调至2~8，考察溶液pH对萃取效率的影响。如图3所示，随着pH升高至中性，萃取效率有微弱的增加趋势。可能是因为灭多威、3-羟基克百威、甲萘威在酸性条件下氨基甲酸酯氮原子质子化，从而导致萃取效率稍低。在pH=8左右，萃取效率迅速下降，可能因为氨基甲酸酯类农药部分水解所致。实验室所用的高纯水pH值为6.8，因此无需额外调节pH。

**图3:**
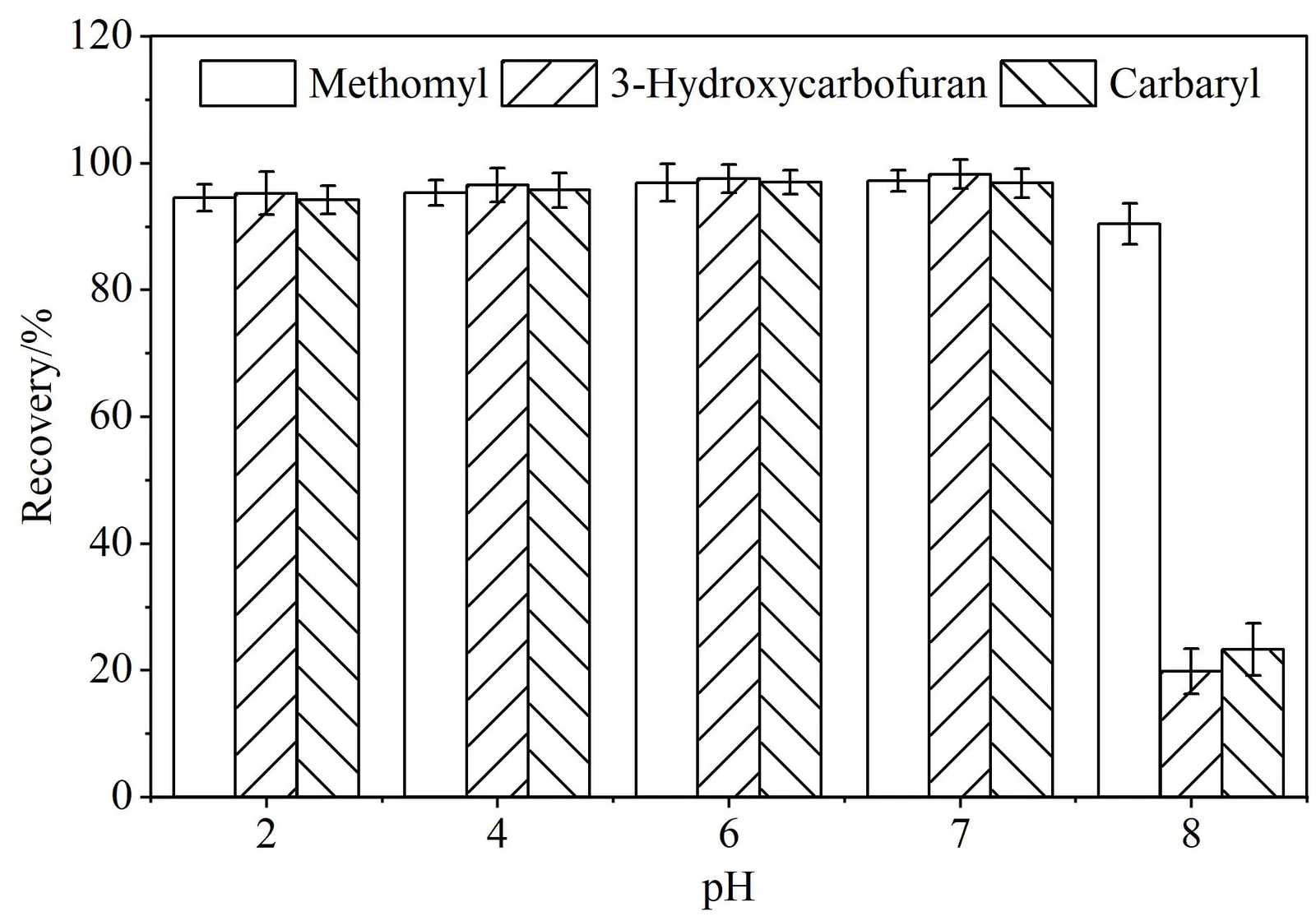
样品溶液的pH对吸附性能的影响（*n*=3）

### 2.3 洗脱条件的优化

分别用甲醇、甲醇-乙腈（1∶1， 体积比）、乙腈作为有机相，考察采用不同洗脱溶剂时氨基甲酸酯类农药的回收率。如图4所示，为了兼顾洗脱与分离需要，我们采用甲醇作为洗脱溶剂中的有机相。

**图4:**
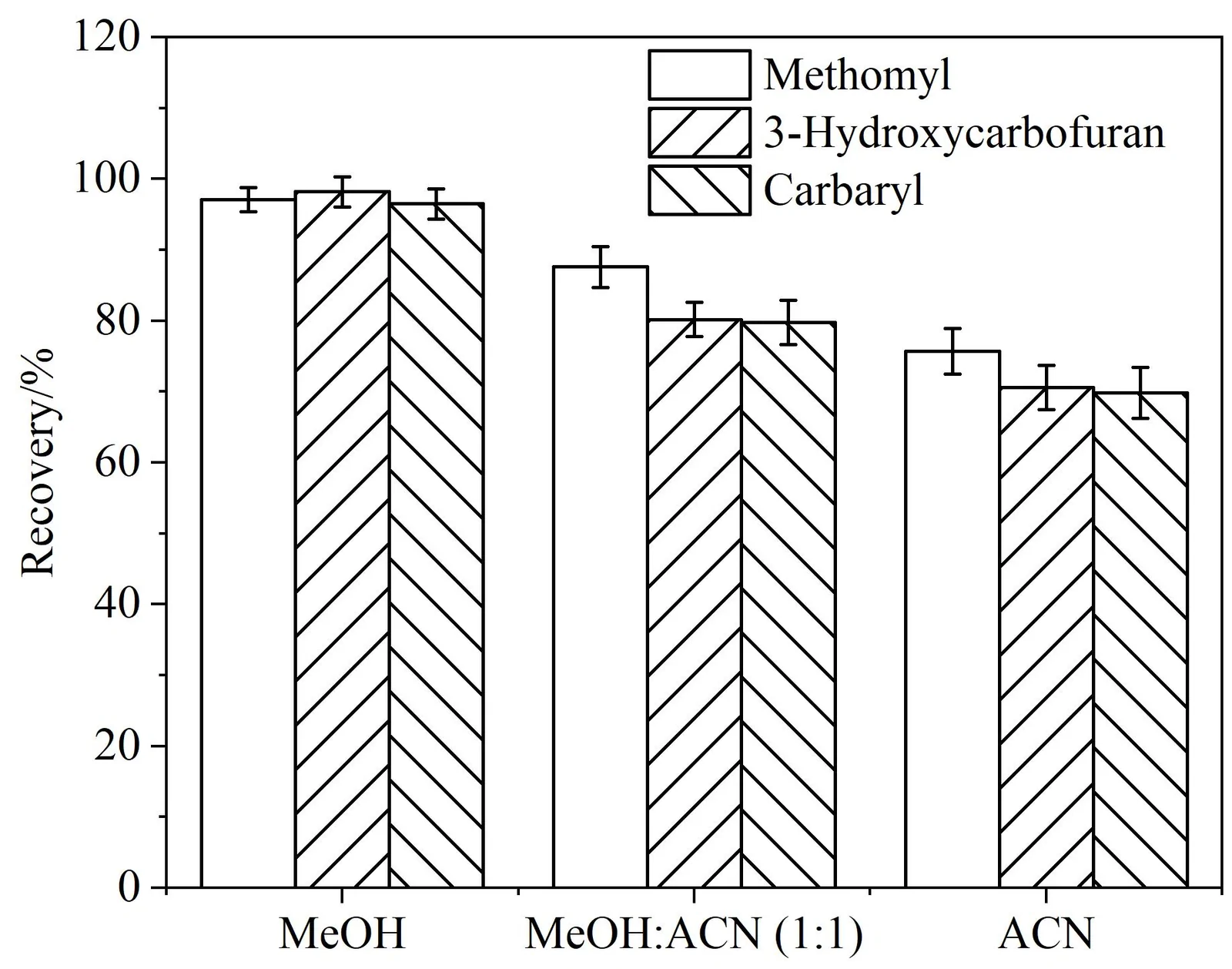
洗脱剂类型对洗脱性能的影响 （*n*=3）

### 2.4 衍生条件优化

#### 2.4.1 衍生液及水解液流速的选择

由于衍生化反应的时间对各氨基甲酸酯类农药的衍生化有较大的影响，为了找到适宜衍生化的流速，分别采用不同的流速（0.2、0.3、0.4、0.5 mL/min）进行优化。如图5所示，随着流速的增大，各氨基甲酸酯类农药的检测回收率逐渐增大后趋于稳定，综合考虑系统压力，我们选择了衍生液及水解液流速均为0.4 mL/min。

**图5:**
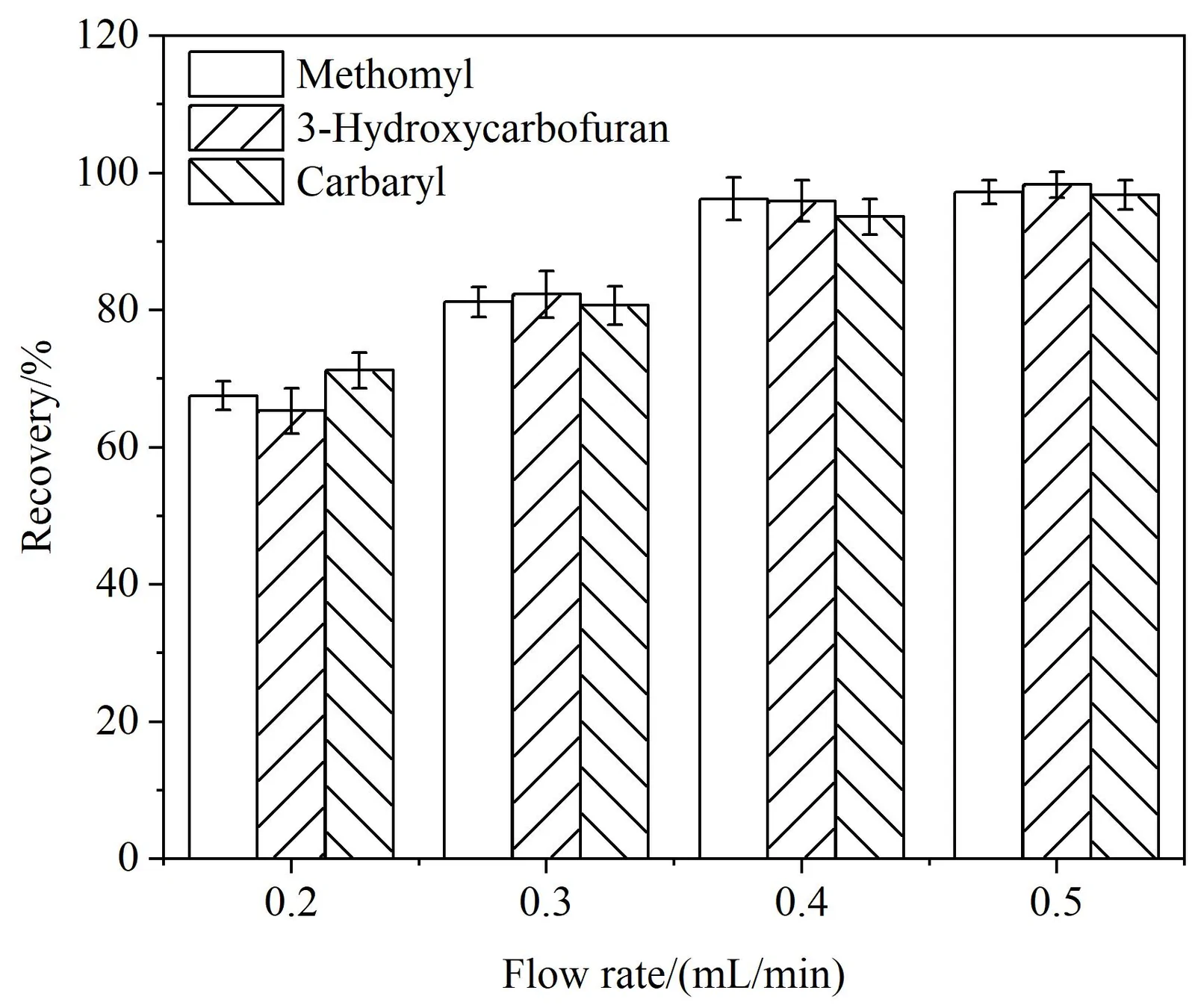
衍生液及水解液的流速对衍生性能的影响 （*n*=3）

#### 2.4.2 水解温度的选择

确定了衍生液及水解液流速均为0.4 mL/min，在其他条件不变的情况下，改变水解反应的温度。如图6所示，分别在70、80、90、100 ℃条件下，进行检测。结果表明，随着温度的升高，各氨基甲酸酯类农药的检测回收率逐渐增大，因此我们选择水解温度为100 ℃。

**图6:**
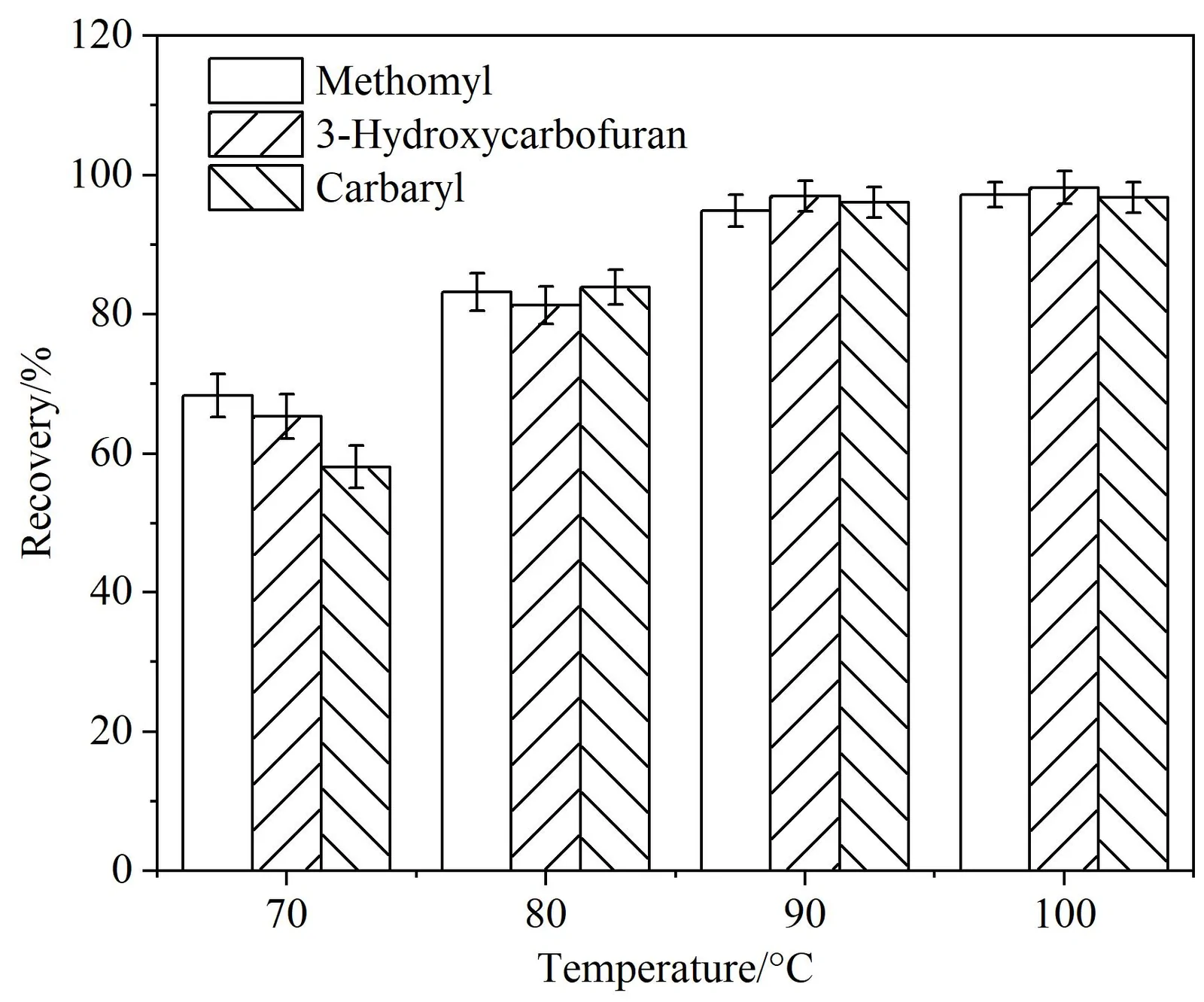
水解温度对衍生性能的影响（*n*=3）

### 2.5 方法分析特性

将系列标准溶液进行液相色谱分析后，以氨基甲酸酯类农药的峰面积为纵坐标（*y*）、对应的质量浓度为横坐标（*x*， μg/L） 绘制标准曲线，采用外标法定量。实验结果表明，灭多威、3-羟基克百威、甲萘威分别在2.0~400、1.0~200、0.5~100 μg/L范围内线性关系良好，相关系数（*R*^2^）均大于0.96（见表2）。氨基甲酸酯类农药的LOD（*S/N*=3）为0.05~0.20 μg/L，LOQ（*S/N*=10）为0.15~0.69 μg/L。对每个标准溶液在1天内平行测定5次以计算日内相对标准偏差（RSD）；每个标准溶液连续测定5天以计算日间RSD。结果显示，所得日内RSD≤2.3%，日间RSD≤4.0%，表明该SPE-HPLC-PCD-FLD系统具有良好的精密度。

**表2:** 3种目标分析物的线性方程、相关系数、线性范围、检出限、定量限、日内RSD（*n*=5）和日间RSD（*n*=5）

Compound	Linear equation	*R*^2^	Linear range/（μg/L）	LOD/（μg/L）	LOQ/（μg/L）	RSDs/% （*n*=5）	
						Intra-day	Inter-day
Methomyl	*y*=4.9*x‒*1.11	0.9979	2.0‒400	0.20	0.69	2.2	3.5
3-Hydroxycarbofuran	*y*=10.9*x*‒0.19	0.9692	1.0‒200	0.10	0.34	2.1	3.6
Carbaryl	*y*=21.7*x*‒1.07	0.9997	0.5‒100	0.05	0.15	2.3	4.0

*y*： peak area of the target compound； *x*： mass concentration， μg/L.

### 2.6 实际样品的测定

将所建立方法应用于3种不同蔬菜样品中灭多威、3-羟基克百威、甲萘威的检测。圆白菜样品、黄瓜样品和芹菜样品中均未检测到灭多威、3-羟基克百威、甲萘威，对样品进行了不同水平的加标回收试验，分析结果如表3所示，蔬菜样品中氨基甲酸酯类农药的回收率为88.2%~96.0%（RSD为1.9%~3.5%）。该方法可应用于实际样品中灭多威、3-羟基克百威、甲萘威的测定。其中一个圆白菜样品加入灭多威、3-羟基克百威、甲萘威标准品（0.2 μg/g）前后的典型色谱图如图7所示。

**表3:** 3种实际样品中氨基甲酸酯的加标回收率 （*n*=5）

Sample	Analyte	Content/（μg/L）	Spiked/（ng/g）	Recovery/%	RSD/%
Cabbage	methomyl	ND	40	88.2	2.5
			200	92.1	3.4
	3-hydroxycarbofuran	ND	40	89.1	3.0
			200	96.0	3.1
	carbaryl	ND	40	87.9	2.9
			200	91.3	3.5
Cucumber	methomyl	ND	40	89.8	1.9
			200	90.5	2.2
	3-hydroxycarbofuran	ND	40	88.9	2.8
			200	92.7	2.6
	carbaryl	ND	40	87.5	3.0
			200	92.2	2.8
Celery	methomyl	ND	40	90.1	3.0
			200	95.5	3.4
	3-hydroxycarbofuran	ND	40	90.2	3.3
			200	94.3	2.8
	carbaryl	ND	40	88.2	3.3
			200	93.0	3.5

ND： not detected.

**图7:**
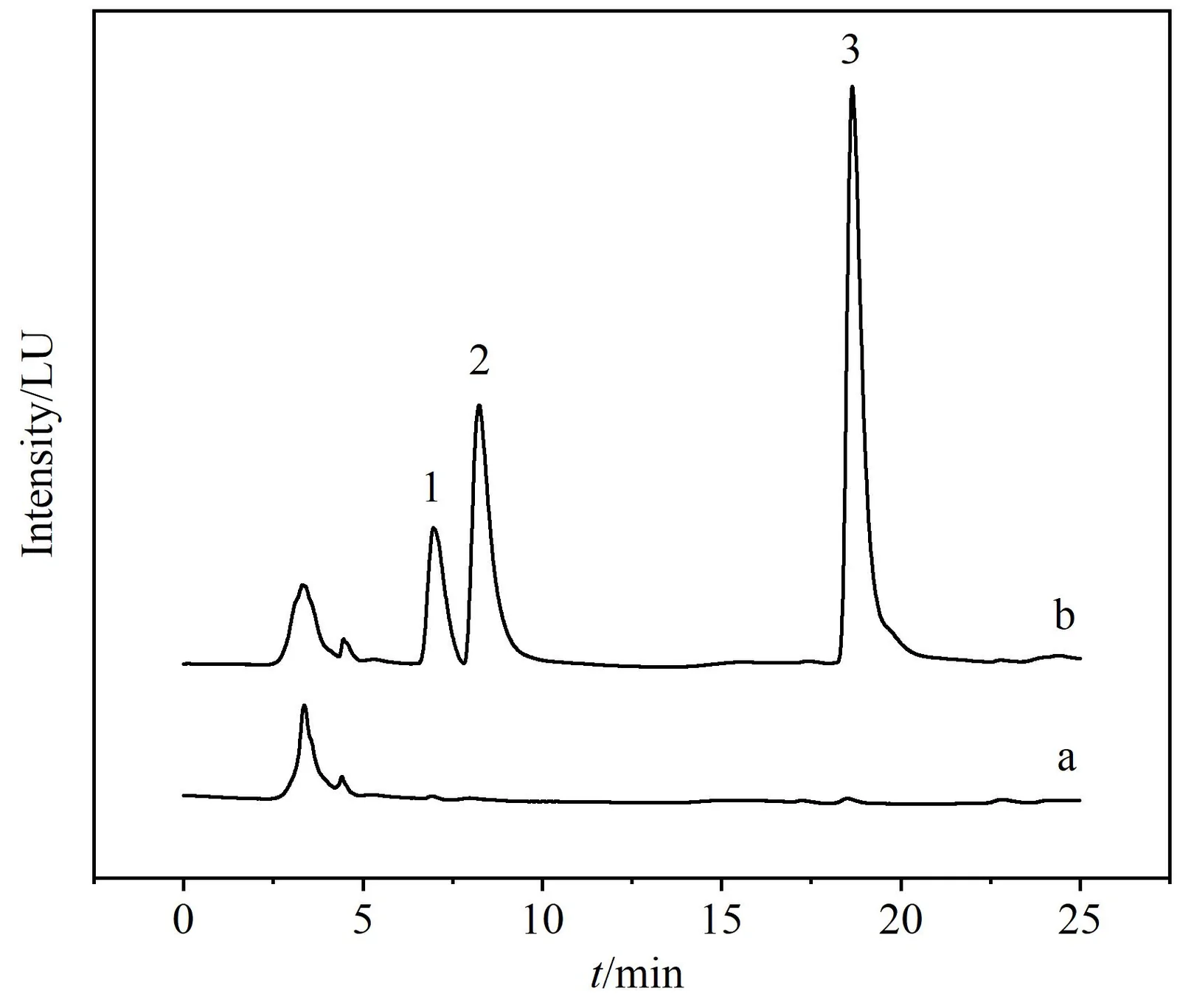
圆白菜（a）空白样品及（b）加标（0.2 μg/g）样品的色谱图

## 3 实验的组织实施及教学反思

### 3.1 实验的组织实施

南开大学仪器分析实验课程近5年来将自主设计实验有机融入教学计划中，与常规实验项目同步进行，形成系统化的教学环节^［5］^。在实验实施过程中，两个常规实验小组合并为10人左右的自主设计实验小组，并推选1名组长，根据成员意见选择课程提供的14种仪器中的一项，随后通过深入学习确定研究方向。常规实验中，教师系统讲授HPLC的基本原理、功能特点及实验方法等理论知识，学生在掌握基础知识后，以小组为单位自主选题、设计实验方案并开展合作探究，完整经历从发现问题、分析问题到解决问题的科学研究全过程。

在具体实施中，学生围绕农药残留检测这一主题，在教师指导下共同确立检测方法、加深对柱前固相萃取与柱后衍生化原理的认识，并完成流路系统的设计搭建。随后，在组长组织下，依次完成蔬菜样品提取液与标准农药溶液的制备、实验系统搭建、性能测试、荧光信号采集、数据分析与结果讨论等环节。

该课题以农药残留检测为切入点，引导学生认识分析化学在保障公众健康与食品安全中的重要作用，增强学生的社会责任感，树立正确的科学价值观，体现了仪器分析实验在食品安全教育中的独特价值。此外，实验中未使用商品化的在线固相萃取和柱后衍生装置，而是为学生提供四元泵、预柱、控温单元、三通、管线等基础部件，鼓励学生在基本高效液相色谱仪的基础上进行功能扩展与系统集成，有效激发了学生的探索热情，显著提升了学生对仪器改造与优化的实践能力。

整个实验过程在教师指导下进行。教师通过“为何需要富集”“为何进行衍生化”“为何选择荧光检测而非紫外信号检测”“为何选择梯度洗脱分离”等启发性问题，引导学生主动查阅资料、设计实验方案。从样品3次平行进样、积分参数的设置，到数据处理、分析方法特征量的计算，增强了学生定量分析的意识。以实际生活问题驱动的探究式学习，充分契合“产学研用”融合的教改方向。这一自主创新实验不仅培养了学生的专业技能，更强化了其社会责任感与创新精神，为其未来科研道路奠定了坚实基础。

### 3.2 实验问题反馈及注意事项

在实验过程中，师生围绕实验原理与操作细节进行深入探讨，共同分析并解决了实验中遇到的一系列问题。例如：衍生试剂的稳定性差，需现配现用；色谱系统扩展模块较多，需注意三通、管线、连接件及密封圈的选型与安装，防止泄漏与超压；系统搭建完毕后，如何进行系统适应性评价与稳定性测试等。通过自主设计实验，学生们普遍体会到液相色谱系统“搭积木”的乐趣，改变了以往“进一针”的刻板印象。

为进一步提高教学质量，计划在以下方面对关键操作进行规范：（1）反应器需预热到温度稳定才能使用，避免基线波动，学生应在教师确认参数正确后再开展实验；（2）预柱使用前需充分平衡，富集后应淋洗杂质；（3）先用标准溶液评价系统性能，并熟悉切换阀操作与系统压力的变化，再循序渐进地进行后续实际样品的检测。

### 3.3 教学反馈

在该教学周期完成后，教师对学生进行了调研，调研结果显示，学生对该自主设计实验表现出较高的成就感与满意度，该在线联用模式的设计，不仅锻炼了动手组装与优化分析模块的能力，也培养了创新思维与问题导向的研究意识。通过实践，学生把握了痕量检测的三大核心策略：样品富集、干扰消除与灵敏检测。

在掌握课堂基本操作的基础上，学生设计并实现了“在线固相萃取-高效液相色谱分离-柱后衍生荧光检测”的方案。该方案显著提高了检测的灵敏度和选择性，让学生们对分析检测全过程有了更加系统、深入的理解。从最初对HPLC认知有限，到通过文献查阅、讨论交流与设计改进，学生的理论知识与科学视野均得到了显著拓展，并对各模块的搭建与衔接、实验方案的设计与优化有了更深刻的理解。该联用方案自动化程度高，大幅缩短了分析时间，在有限学时内呈现出更完整的知识讲解与原理分析。

尽管实验初期遇到了诸多困难，但在教师指导与团队协作下均得到有效解决，使学生深刻体会到科研探索的乐趣与成就感。例如在实验初期选择衍生条件时，学生们对流动相的种类、比例以及流速参数缺乏选择思路，经过与老师讨论后多次尝试，最终优化出了最合适的洗脱条件。该案例说明，仪器分析自主创新实验不仅强化了学生们的操作技能与数据分析能力，还有效激发了他们的科研兴趣和解决问题的能力，为将来他们从事分析化学以及相关领域的研究打下了扎实的基础。学生普遍反映，通过自主设计实验，他们对实际问题的解决路径有了更深入的理解，对未来的科研工作充满信心。

实验在实现教学目标的同时，也发挥了良好的育人功能。将HPLC实验与食品安全这一社会热点相结合，学生们更加直观地认识到分析化学技术的社会价值。这些元素的有机融入，培养了学生们的社会责任感与历史使命感，为培养兼具服务社会意识和国际视野的高素质人才提供了有力支撑。

## 4 结论

在师生的共同努力下，本实验成功实现了柱前在线固相萃取、高效液相色谱分离与柱后衍生荧光检测的在线集成，建立了氨基甲酸酯类农药残留的高灵敏度分析方法，不仅丰富了课程内容，也提升了学生自主学习和解决实际问题的能力。教师在指导学生实验过程中也不断优化教学策略，有效提升了教学质量。

仪器分析实验课程引入自主设计实验环节后，整体更加完善，学生对HPLC的原理以及众多组成模块的作用有了更透彻的理解，实现了从被动接受基本知识到主动探究学习模式的转变，对于其他仪器分析方法的学习具有借鉴意义。学生在掌握了氨基甲酸酯类农药的衍生技术及具体操作流程的基础上，对实验的工作原理和方法优化建立了系统性认知，在食品安全的研究背景下提升了科学素养与社会责任感，为将来科研能力的培养奠定了坚实的基础。

该自主设计实验方案构建了一个紧密结合社会需求的综合性教学模式，可作为仪器分析实验教学改革的典型范例，具有一定的推广价值。
